# Stable Morphology, but Dynamic Internal Reorganisation, of Interphase Human Chromosomes in Living Cells

**DOI:** 10.1371/journal.pone.0011560

**Published:** 2010-07-13

**Authors:** Iris Müller, Shelagh Boyle, Robert H. Singer, Wendy A. Bickmore, Jonathan R. Chubb

**Affiliations:** 1 Division of Cell and Developmental Biology, College of Life Sciences, University of Dundee, Dundee, United Kingdom; 2 MRC Human Genetics Unit, Institute of Genetics and Molecular Medicine, University of Edinburgh, Edinburgh, United Kingdom; 3 Department of Anatomy and Structural Biology, Albert Einstein College of Medicine, Bronx, New York, United States of America; Centre National de la Recherche Scientifique, France

## Abstract

Despite the distinctive structure of mitotic chromosomes, it has not been possible to visualise individual chromosomes in living interphase cells, where chromosomes spend over 90% of their time. Studies of interphase chromosome structure and dynamics use fluorescence in-situ hybridisation (FISH) on fixed cells, which potentially damages structure and loses dynamic information. We have developed a new methodology, involving photoactivation of labelled histone H3 at mitosis, to visualise individual and specific human chromosomes in living interphase cells. Our data revealed bulk chromosome volume and morphology are established rapidly after mitosis, changing only incrementally after the first hour of G1. This contrasted with the behaviour of specific loci on labelled chromosomes, which showed more progressive reorganisation, and revealed that “looping out” of chromatin from chromosome territories is a dynamic state. We measured considerable heterogeneity in chromosome decondensation, even between sister chromatids, which may reflect local structural impediments to decondensation and could potentially amplify transcriptional noise. Chromosome structure showed tremendous resistance to inhibitors of transcription, histone deacetylation and chromatin remodelling. Together, these data indicate steric constraints determine structure, rather than innate chromosome architecture or function-driven anchoring, with interphase chromatin organisation governed primarily by opposition between needs for decondensation and the space available for this to happen.

## Introduction

Unlike the characteristic morphology of condensed mitotic chromosomes, the structure and organisation of specific individual chromosomes in the interphase nuclei of living cells is not known.

Fluorescence in situ hybridization (FISH) on fixed cells has allowed visualisation of individual loci, chromatin domains and whole chromosomes in the nucleus [1]. However, FISH is not applicable to living cells and so dynamic aspects of chromatin organisation can only be inferred from snapshots. There is also the concern that the fixation and DNA denaturation steps of FISH damage chromosome structure. Standard fixation uses methanol-acetic acid or formaldhyde. The former operates by dehydration and is particularly damaging to 3D architecture, causing a flattened nucleus and damaged chromosome morphology [2], [3]. Whilst formaldehyde is thought to be more satisfactory, the tendency of this fixative to trigger retraction of cells' cytoskeletal protrusions urges for caution [4]. High temperature formamide denaturation steps are necessary to open chromatin and the DNA double-helix to allow probe access for hybridisation and the balance between probe access and major structural damage must be carefully monitored. Finally, c*ot1* DNA is usually added with the probe to quench hybridisation to highly repetitive sequences, therefore only the low copy number part of the chromosomal sequence is visualised, which may not represent the properties of the entire sequence [5].

Approaches for visualising and monitoring individual loci in living cells have been developed, such as the targeting of GFP to integrated arrays of bacterial operator repeats [6]. Techniques also exist for the random labelling of chromosome sub-domains, using replication incorporated fluorescent dNTPs [7] or photobleaching/photoactivating fluorescently tagged histones [8], [9], [10]. But techniques for monitoring the morphology and dynamic organisation of specific chromosomes in living cells are lacking.

Following decondensation after cell division, chromosomes occupy discrete territories in the interphase nucleus [11]. Interphase chromosomes can display radial organisations determined by chromosome size or gene density [12], [13], [14] and favour cell-type specific chromosomal neighbourhoods [15]. Chromatin domain position is established early in G1 [16], [17] and appears to be stable during most of interphase [9]. Long range movements of chromosomal domains in interphase nuclei are seen rarely [18], [19] with most chromatin confined to submicron regions and undergoing only limited diffusion [20], [21], [22]. Interest in chromosome architecture and dynamics has been revitalised by demonstrations that chromosome position and nuclear organisation contribute to gene regulation [23], [24], [25], [26].

We have therefore developed a labelling strategy for observation of single chromosomes in living interphase nuclei. The chromatin label is photoactivateable GFP (PA GFP) [27] fused to histone H3. This has given us, for the first time, the possibility to observe chromatin decondensation at the single chromosome level and to study whole chromosome structure and dynamics in interphase nuclei. In combination with the fluorescent tagging of a specific locus, we demonstrate that bulk chromosome architecture is stabilised soon after mitosis but that positioning of individual loci relative to chromosome territories can be more progressively established. Chromosome structure is surprisingly resistant to impairment of nuclear functions. Our data support a view where chromosome architecture describes the balance between necessary chromatin decondensation and accessibility against the limitation of available space by the lamina, nuclear compartments and other chromosomes.

## Results

### Labelling single chromosomes in living cells

**Figure 1:**
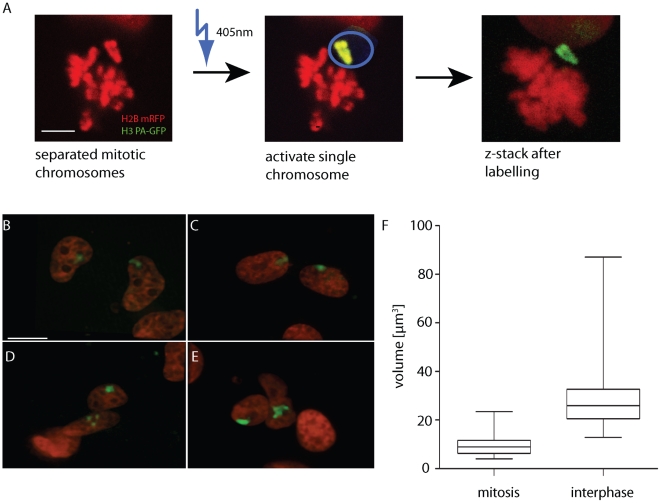
Labelling individual human interphase chromosomes in live cells. A) Activation of H3 PA-GFP (green) on a single chromosome in mitosis. Using H2B mRFP, mitotic cell with separated chromosomes were identified and separated chromosomes were scanned using a 405 nm laser. A z-stack was used to confirm single chromosome labelling (right image, projection of 3D stack, 5 µm bar). B–E) Sample maximal projections of different pairs of daughter nuclei (red) showing single interphase chromosomes (green). Bar 15 µm. Images captured between 1 and 4 hours after mitosis. F) Quantification of decondensation of mitotic chromosomes into 4 h interphase chromosomes. Box plots show data range of chromosome volumes (µm^3^), the two mid-quartiles, and the median. Mitotic chromosomes n = 20, interphase n = 39.

To visualise single interphase chromosomes, we photolabelled chromosomes during mitosis, when they are condensed and distinct (Figure 1A). The histone H3.1 variant (HIST1H3A) was chosen for labelling as it shows very slow turnover on chromatin, with approximately 80% of incorporated H3.1 showing no turnover after incorporation during S-phase [28]. In comparison, H2B has a major (40%) fraction with a half time for turnover of around 2 hours, and the replacement H3 variant, H3.3 was also expected to be more dynamic [29]. We initially tried photobleaching a H3-GFP fusion, but the extensive laser treatment meant chromosomes often moved before labelling was complete, and cells arrested in mitosis because of photodamage. Therefore, we generated human HT-1080 cell lines stably co-expressing Histone 2B mRFP (H2B-RFP) to identify cells in mitosis, and Histone H3 PA-GFP (H3-PA-GFP) fusions (Figure 1A). HT-1080 cells were selected as their chromosomes lack the translocations commonplace in other transformed human cell lines [30].

During congression to the metaphase plate, chromosomes displayed different times of convergence, meaning chromosomes were often transiently separated from the main chromosome mass. A region around separated chromosomes was scanned 6 times with a 405 nm laser on a confocal microscope (Figure 1A). This caused activation of the fluorescence of the photoactivateable GFP. Afterwards a 3D stack of the mitotic cell was captured to check for labelling of a single chromosome. Incompletely labelled chromosomes, or cells where labelling could be detected on other chromosomes, were not imaged further. To increase the overall number of mitotic cells and therefore the chance of finding separated chromosomes, the cells were incubated with Monastrol, a reversible Eg5 kinesin inhibitor, which arrests cells in mitosis [31]. 3D images of the two daughter chromosomes were captured at time periods after mitosis (Figure 1B–E). The H3 PA-GFP signal was stable for up to 5–6 hours after labelling, before a combination of turnover and activation of H3 PA-GFP in the rest of the nucleus during imaging restricted further capture. To keep background activation at a low level, imaging of interphase chromosomes was performed on a wide-field station designed for low bleaching and long term imaging of photosensitive cells [32], [33], [34]. Labelled chromosomes were re-identified on this imaging station with the aid of microscopy chambers with calibrated grids.

### Decondensation at the single chromosome level

Interphase chromosomes showed tremendous morphological diversity. There were compact globular structures (53.8% of total) (Figure 1C right chromosome and 1E left chromosome), dumbbell-like (12.8%) (Figure 1B right chromosome and Figure 1C left chromosome), branched structures (30.8%) (Figure 1B left and Figure 1E right) and chromosomes where the mass was dispersed around nucleoli (2.6%) (Figure 1D lower nucleus).

Measuring the volumes of 39 chromosomes 4 hours after mitosis, a high variability in size was observed (Figure 1F). The volume ranged from 13 µm3 for the smallest chromosome to 87 µm3 for the largest, with a mean of 29 µm3. Chromosomes engaged a proportion of the nuclear volume from 1.8–12.0%, with mean 3.7% occupancy. In line with our measurements, human chromosome 1 spans 247 Mb [35] and holds 8% of total nuclear DNA per chromosome.

Comparing sister chromosomes in interphase revealed similar morphology and nuclear position in some daughter pairs, but considerable heterogeneity between other daughter chromosomes (Figure 1B–E). Variability in sister chromosome volume ranged between 4.9% and 68% with a mean of 20.4%. These differences reflected the space single chromosomes occupied, and were independent of nuclear volumes. It is not clear whether all chromosomes can display sister variability, or just a subset. Chromosome 11 clearly shows considerable heterogeneity, as described below. Quantitative differences in labelling between sister chromatids might contribute to the observed heterogeneity in volume, but as incompletely-labelled chromosomes (assessed at mitosis) were excluded from our analysis, the large scale morphological differences apparent between some decondensing chromatids (such as Figure 1D,E) are unlikely to be due to this caveat.

Our method allows direct comparison of single mitotic chromosomes with their daughters in interphase. The average mitotic chromatid had a volume (half the chromosome) of 10 µm^3^ with a range of 4 to 23 µm^3^. The mean increase in chromosome volume over the first 4 hours of interphase was over 300% (Figure 1F). We were surprised by the large variability in decondensation in different cells. The largest mitotic chromosome (23.45 µm^3^) showed only a slight (0.4%) increase in volume, generating an average-sized interphase chromosome. While 2 medium-sized mitotic chromosomes (around 12 µm^3^) showed increases of 430% up to 720% in size and were two of the biggest interphase chromosomes detected. This individual behaviour of different chromosomes might be due to different gene densities or diverse transcriptional or epigenetic states of individual chromosomes [36]. Variability in decondensation of sister chromatids indicates stochastic chromosome-wide influences, such as molecular crowding, also contribute greatly to diversity of decondensed size and morphology.

### Live imaging of defined chromosomes in interphase

**Figure 2:**
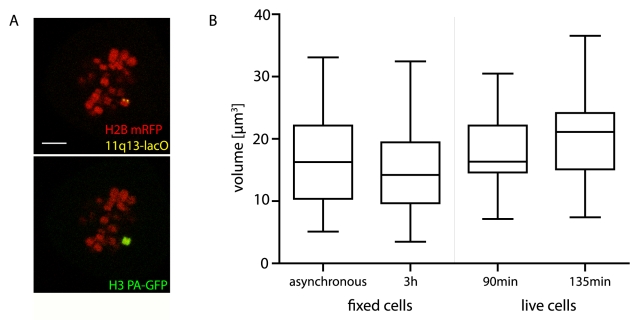
Human chromosome 11 visualised in live interphase cells. A) Activation of chromosome 11, defined by visualisation of a lacO integration into 11q13 in HT-1080 cells. 11q13 marked by YFP-lacI, which binds the lacO repeats (yellow spot, see top panel). Bar 5 µm. B) Box plots of volumes (µm^3^) of chromosome 11 in living and fixed cells. 3D stacks of live cells were performed 90 (n = 18) and 135 (n = 17) min after mitosis. FISH was carried out on cells fixed from asynchronous cultures (n = 58) or 3 h (n = 65) after release of overnight synchronisation with cdk1 inhibitor.

To investigate the structure of a defined chromosome in interphase we took advantage of an HT-1080-derived cell line with a lacO repeat inserted at 11q13 (11q13-lacO HT-1080)[16], [23]. To visualise the lacO repeat, a YFP-lacI fusion protein was stably expressed together with H2B mRFP and H3 PA-GFP. The probability of finding a separated chromosome 11 in mitosis was very low, even in the presence of Monastrol, so as an alternative, we used the Cdk1 inhibitor RO-3306 [37] which arrests cells at the G2/M border. After removal of RO-3306, cell number increased by 53% (3 replicates, 1151 cells at G2/M, 1757 cells at 3 h) in the next 3 hours, indicating around half of cells accumulated at G2/M during overnight synchronisation. Single chromosomes 11 were photo-activated (Figure 2A) and monitored. The mean volume of chromosome 11 was 17.7 µm^3^ at 90 min after mitosis and 20.3 µm^3^ by 135 min, indicating chromosome 11 is slightly smaller than the mean chromosome volume. With a size of 134.5 Mb for chromosome 11, we therefore estimate human chromatin packing in living cells at 0.15 µm^3^/Mb. As with randomly labelled chromosomes, chromosome 11 also showed a high variability in volume, with sister chromosomes showing differences of up to 78.9%. However, the mean difference in volume between sister chromosomes (8 pairs) at 135 minutes was half the difference between volumes of randomised chromosome 11 pairs, suggesting effects on chromosome morphology inherited through cell division.

Specific labelling of chromosome 11 allowed comparison of living cell data with FISH data from formaldehyde fixed cells. FISH experiments using a probe for the entire chromosome 11 were performed on the same 11q13 cell line, grown under the same conditions. As live imaging was performed on predominantly G1 cells, we wished to minimise cell cycle differences in volume data. Therefore in addition to assessing chromosome 11 volumes from fixed asynchronous cells, we measured cells fixed 3 h after removal of the cdk1 inhibitor, reflecting an enriched early G1 population. There was no difference in measurements of chromosome 11 volume between fixed asynchronous cells and live cells 90 minutes (p = 0.5867) and 135 minutes (p = 0.1067) after mitosis (Figure 2B). For fixed cells, mean volume was 17.1 µm^3^ for asynchronous cultures (SD 7.8), compared to 20.3 µm^3^ in living cells (135 min, SD 7.0). The large variance in volume data from FISH was not simply a consequence of variability in hybridisation efficiency, as live cell measurements had a similar variance. A small difference in chromosome volume could be detected between live cells 135 minutes after mitosis and RO-3306-treated fixed cells (p = 0.008). In addition to any structural effects of FISH, we attribute this difference to uneven passage through mitosis after removal of synchronisation, hence some nuclei would have been fixed during decondensation.

The absence of a large difference in chromosome volume (measured by FISH) between G1 (RO-3306-synchronised) and asynchronous populations, is intriguing. Superficially, this suggests volumes of replicated and unreplicated chromosomes are similar. However, measurements of total nuclear volume (defined by H2B-RFP) suggest this is not the case. By long term imaging H2B-RFP defined nuclei, we found the nuclear volume 4 hours after mitosis (mid G1) was almost exactly half (48.7%, n =  15 daughter nuclei) the premitotic volume. Constancy of volume may instead reflect a short G2 and rapid decondensation in early G1, so that fully replicated or condensed interphase chromosomes are present for a small time only.

### Establishment and maintenance of interphase chromosome morphology

**Figure 3:**
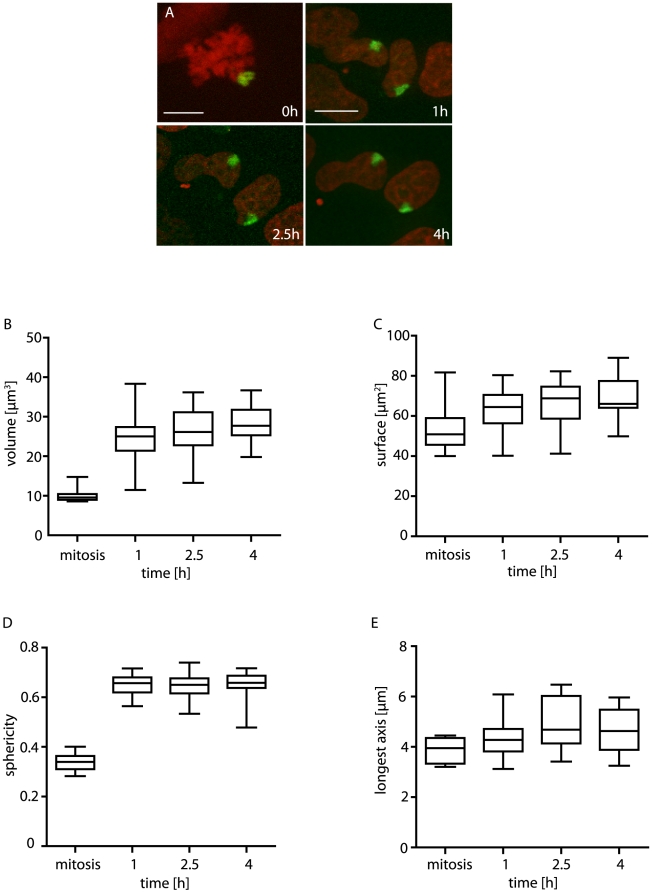
Chromosome volume and morphology are defined within the first hour after mitosis. A) Decondensation of a single mitotic chromosome (upper left, bar 5 µm) into two daughter nuclei in interphase (1, 2.5 and 4 hours after mitosis, bar 10 µm). Images are maximal projections of 3D stacks. B–E) Box plots showing changes of volume (µm^3^) (B), surface area (µm^2^) (C), sphericity (D) and longest axis (µm) (E) of chromosomes at mitosis and 1 h, 2.5 h and 4 h into interphase. All data sampled from the same movies, of 6 mitotic chromosomes and their 12 interphase descendents.

Little is known about how interphase organisation of chromosome architecture is established in the cell cycle. We therefore analysed the dynamic morphological behaviour of single activated chromosomes at 1, 2.5 and 4 hours after the completion of mitosis (Figure 3A).

Chromosome morphology was established within the first hour after mitosis. Expansion of randomly labelled chromosomes by 250% occurred within the first hour after mitosis, and increased only slightly thereafter (Figure 3B). The decondensation of chromosome 11 was also largely complete soon after mitosis, with only a slight increase in mean volume (17.7 to 20.3 µm^3^) occurring between 90 and 135 minutes after mitosis (Figure 2B). The doubling time for HT-1080 cells is approximately 1 day. G1 makes up one third of the cell cycle in HT-1080 cells [38], so although cell cycles are naturally highly heterogeneous in length, clearly the major chromosome volume expansion occurs very early in G1. The literature appears to have a solid consensus that large chromatin domains are relatively immobile in interphase [8], apart from very early G1 [9]. In agreement with this, and apparent in the images in the present study, we observed no large scale redistributions of chromosomes, despite clear changes in nuclear orientation and cell positioning.

Chromosome surface area provides a measure of morphology changes during decondensation (Figure 3C). To approximate mitotic chromatid surface areas, measured values for mitotic chromosomes were halved. Only a mild increase in surface area occurred during decondensation, and this difference may be attributed to the small variability in area measurements between the confocal and widefield imaging stations used for mitosis and interphase, respectively (see methods).

To identify changes in shape and structure of the chromosomes we calculated their sphericity (Ψ) using Ψ =  (π^1/3^*(6V)^2/3^)/A (with V =  volume and A = surface area). A Ψ of 1 is a perfect sphere. The more Ψ approaches zero, the less spherical the object. Interphase chromosomes are more spherical (Ψ = 0.65) than mitotic chromosomes (Ψ = 0.3; Figure 3D). The greatest change in sphericity occurred in the first hour of interphase and was stable thereafter (Figure 3D). There was no significant change in length of longest axis between mitotic and interphase chromosomes (Figure 3E).

Several contributions to stability of chromosome architecture have been considered. The presence of multiple genes at sites of transcription (transcription factories) has been postulated to act as a stabilising force on chromosome architecture [39] and inhibition of transcription can cause some retraction of chromatin loops [40]. Self-organisation of inactive chromatin may also contribute [41], [42]. Although controversial, the idea of a nuclear matrix, often conflated with the idea of chromosome scaffolds, could explain chromosome stability [43]. An additional idea is that stability is actively maintained by ATP-dependent chromatin remodelling enzymes, an idea supported by the strong effects on nuclear-wide chromatin condensation caused by blocking ATP synthesis [44].

**Figure 4:**
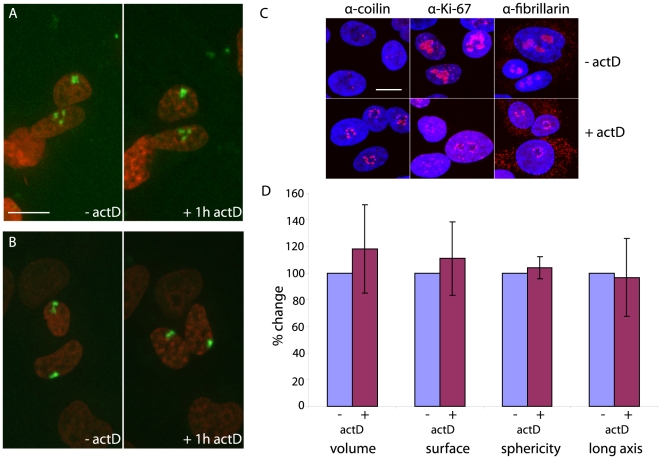
Resistance of chromosome territory volume and morphology to transcription inhibition. A,B) Two examples of single chromosomes (green) in the interphase nucleus (red) before (1.5 h after cell division, left picture) and after treatment for 1 h with 1 µM actD (right picture). Bar 12 µm. C) Typical disruption of nucleolar components upon transcription inhibition by 1 h treatment with actD. Antibody staining (red) against 3 different nucleolar proteins (coilin, pKi67 and fibrillarin). Nuclei were stained using DAPI (blue). Bar 10 µm. D) Volume, surface area, sphericity and longest axis of single chromosomes (n = 14) before (blue bar) and after 1 h actD treatment (red bar). The values of the chromosomes before the treatment were set independently to 100% and data for treated chromosomes was calculated relative to these.

To address to what extent transcription stabilises chromosome structure we used the transcriptional inhibitor actinomyocinD (actD) [45]. 1 µM actD strongly inhibited the ability of HT-1080 cells to transcribe, as assessed by cellular incorporation of the uridine analogue 5-fluorouridine into nascent RNA (Figure S1A). Effects of actD on nuclear structure were observed using immunofluorescence with nucleolar antibodies (Figure 4C) which showed typical disruption of nucleolar structure by drug treatment [46]. If transcription maintained chromosome structure, a transcription inhibitor would be expected to alter chromosome volume and morphology, leading to a more condensed state. The overall structure of the dispersed chromosome in Figure 4A did not change after being exposed to actD, even as the nucleolus diminished in size. A few chromosomes did show changes. The upper chromosome in Figure 4B initially showed a diffuse extension before retraction after inhibition of transcription. However, averaged over all the labelled chromosomes, inhibition of transcription led to no significant quantitative changes of volume, surface area, sphericity or longest axes of randomly labelled chromosomes (Figure 4D, p values all >0.35). Examples where transcription did appear to maintain chromosome structure may reflect particularly gene dense chromosomes or chromosome segments [36].

**Figure 5:**
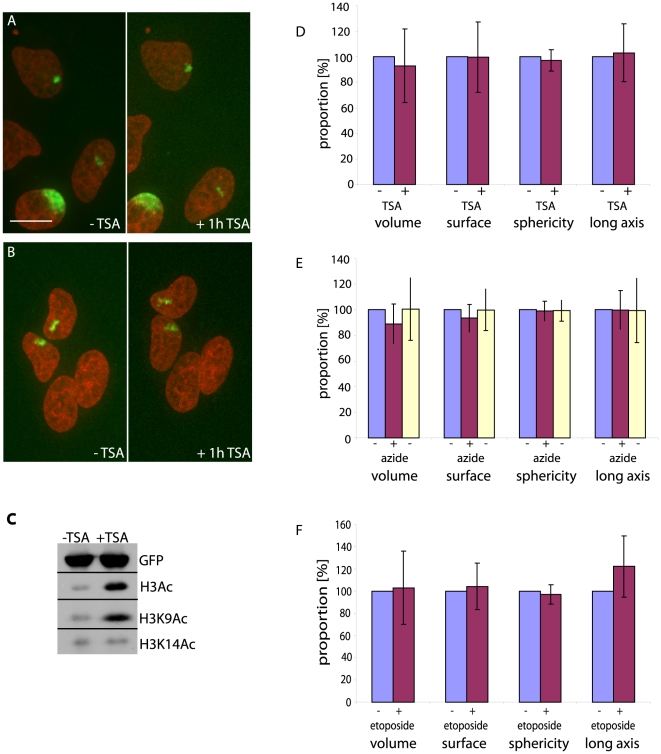
Chromosome territory structure is resistant to inhibition of HDACs, ATP synthesis and Topoisomerase II. A,B) Example images of single chromosomes after TSA treatment. Projections of 3D stacks shown 1.5 h after mitosis (before treatment) and after 1 h incubation with 10 nM TSA. Bottom cell in A was marked during activation of the mitotic chromosome. Bar 12 µm. C) Western blots to detect histone acetylation levels before and after 1 h TSA treatment using antibodies against H3Ac. Loading checked using a GFP antibody to assess H3 PA-GFP. The same extracts were loaded for all blots, but run separately. D) Data from 13 daughter chromosomes were used to calculate changes in volume, surface area, sphericity and longest axis before (blue bar) and after 1 h of TSA (red bar). Values for chromosomes before TSA treatment were set to 100% and data for treated chromosomes calculated as a proportion of this. No changes in volume, surface area, sphericity and longest axis of the chromosomes were observed with either ATP depletion by azide and 2-deoxyglucose (n = 30) (E) or topoisomerase II inhibition with etoposide (n = 7) (F). For ATP depletion, images were also captured after removal of treament (yellow).

To disrupt potential roles for inactive chromatin in the maintenance of chromosome architecture, we treated cells with the histone deacetylase inhibitor Trichostatin A (TSA). This caused an increase in general levels of histone H3 acetylation and H3K9 acetylation (Figure 5C). Hyperacetylation of histones has been suggested to result in chromatin opening [47]. However, TSA induced no significant changes in chromosome morphology in living cells (Figure 5A,D; p values >0.2). Both sister chromosomes from one mitosis appeared to decondense after TSA treatment (Figure 5B) although these changes were lost in population averages. Effects on specific chromosomes or chromosome segments cannot be excluded [48].

To address the requirement for ATP for maintenance of chromosome architecture, we treated cells with 2-deoxyglucose together with sodium azide, to inhibit ATP synthesis [49]. Effects of ATP depletion on chromatin could be observed directly on H2B-RFP distribution as differences in chromatin structure within the nucleus became more contrasted (Figure S1B). In particular, heterochromatin around nucleoli became more prominent. Removing the treatment reversed these effects. However, these changes on local chromatin density did not appear to strongly affect chromosome volume or morphology. A Wilcoxon test found significant differences in surface area and volume between chromosomes before and after treatment (p values: 0.0.0019 and 0.0038 respectively). However, these effects were heavily dependent upon pairing of values and not apparent in means (Figure 5E). We also addressed the requirement for Topoisomerase II, a major component of nuclear matrix preparations, for maintenance of chromosome architecture. Topoisomerase II inhibition can also induce DNA damage, which can be observed in HT-1080 cells as nuclear-wide H2AX phosphorylation in all cells after one hour of this treatment (Figure S1C)[50]. Despite these effects, 50 µM etoposide had no measurable effect on chromosome territory structure (Figure 5F; p all >0.15).

### Dynamics of chromatin looping

The chromosome territory defined by FISH with chromosome paints does not define the limits of spread of chromatin from that territory. Not only do chromosomes intermingle [51], they can also project chromatin beyond the limits defined by chromosome-specific FISH probes [52], [53], [54], [55]. Inactive alleles (defined by RNA FISH) are found preferentially within chromosome territories, whereas actively transcribing alleles, and those associated with transcription factories (defined by immuno-DNA FISH) are found both inside and outside of territories [56], [57]. “Looping out” is only seen in a proportion of alleles in the cell population and it is not known whether this reflects a dynamic movement in and out of the territory, or differences between the behaviour of alleles in the population. The process of looping out has also only been observed in fixed cells, raising persistent concerns it may be an artefact of FISH protocols.

Sequences from the gene dense 11q13 region have been demonstrated to loop out from the chromosome 11 territory in 60–70% (depending on fixation) of territories in human lymphoblastoid cell lines [40]. 11q13 is the same region where lacO arrays are inserted in the 11q13-lacO HT-1080 cell line. The precise integration site is unclear, but lies within chr11:64,800,000–65,600,000 bp (using the NCBI35/hg17 build of the human genome) [23]. 11q13 is a RIDGE, a region of high density of high gene expression in many cell types [58] and is a region of the human genome that supports high expression of integrated transgenes [59]. Earlier expression studies indicate many of the genes flanking the lacO array are expressed in this clone [23].

**Figure 6:**
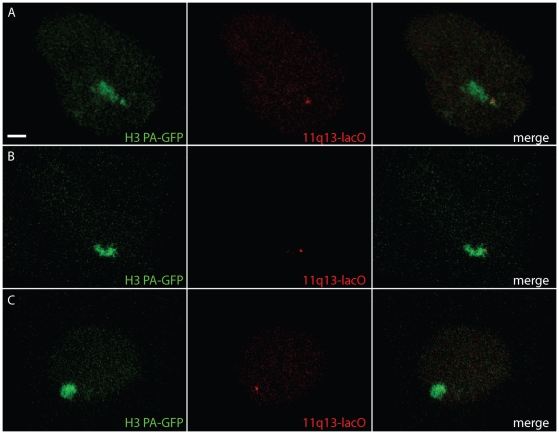
Imaging a specific locus and its chromosome territory in living human cells. Representative images of three different nuclei revealing the relative distribution of chromosome 11 and the 11q13 locus 135 minutes post-mitosis. Maximal projections of 3D stacks of chromosome 11 (green, left) and lacO repeats (red, middle) or merged (right) at 11q13 in 3 different positions: outside main territory (A), edge of territory (B) and inside territory (C). Bar 10 µm.

**Figure 7:**
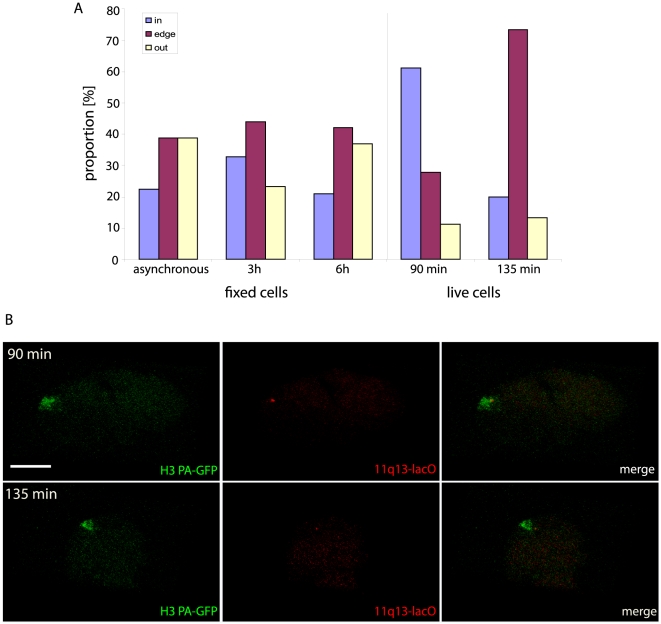
Dynamics of 11q13 projection from chromosome 11. **A**) Localisation (inside, edge or outside) of the 11q13 relative to chromosome 11 territory. Analyses were performed either in live cell experiments at 90 min (n = 18) or 135 min (n = 17) after cell division or in FISH experiments on asynchronous (n = 139) or synchronized cells 3 h (n = 134) or 6 h (n = 138) after release. Localisation was identified as inside the territory (blue), edge of the territory (red) or outside the territory (yellow). The 11q13-lacO spot was only “outside” if it was totally detached from the territory surface. B) Dynamic projection of 11q13. Images of 11q13 (red) localised inside the chromosome 11 territory (green) at 90 min and outside at 135 min after mitosis. Bar 10 µm.

Using the 11q13-lacO HT-1080 cell line, we were able to observe 11q13 and the chromosome 11 territory together in living cells and assess whether looping out can be detected without fixation. FISH experiments on asynchronous fixed 11q13-lacO HT-1080 cells, revealed 39% of lacO hybridisation signals detected outside the territory. In living cells, we observed examples for three different states of lacO repeat position: “out” (Figure 6A), “edge” (Figure 6B) or “in” (Figure 6C) the chromosome territory. The data from 18 lacO spots at 90 min after cell division showed 2 spots outside the territory, with 5 at the edge and 11 inside (Figure 7A).

In living cells 135 min after mitosis there were still two 11q13 spots outside the chromosome volume, but we detected a strong shift of spots from inside the territory (69% at 90 min) to the edge (73% at 135 min) (Figure 7A). An outward movement of 11q13 was also measured using FISH on synchronized cells. 3 h after release of the cell cycle block, when 50% of cells would be in early G1, only 23% of all spots were outside the chromosome territory compared to 39% for asynchronous cells. 6 hours after release of synchrony, the proportion outside the territory had increased to 37%. Together, these data indicate looping out of loci from the bulk chromosome mass is progressive during G1.

These dynamics of 11q13 relative to the territory edge were clearly observed in live images. The 11q13 locus in Figure 7B was initially on the edge of the chromosome (90 minutes) before moving away from the territory by 135 minutes. Unlike the rapid and stable establishment of chromosome territory architecture, looping is progressive and dynamic, perhaps reflecting the gradual activation of transcribed genes in the projected chromatin. We observed 2/18 spots outside the territory at both 90 minutes and 135 minutes, however, the 2 projected 11q13 loci were from different cells at the different time points, indicating looped-out chromatin can also be retracted.

## Discussion

We have presented a new technology for the analysis of interphase chromosome structure and dynamics in living human cells. The study provides basic quantitative measures for physical properties of chromosomes in living cells. Our data reveal that chromosome volume and morphology are established rapidly after mitosis, changing only marginally after the first hour of G1. This contrasts with the behaviour of a locus on chromosome 11, which appeared to have a more gradual, progressive spatial reorganisation. Bulk chromosome morphology and volume showed tremendous resistance to inhibitors of various nuclear functions, such as transcription, histone deacetylation and chromatin remodelling, although local chromatin changes and effects on nuclear compartments could clearly be detected. We also measured heterogeneity in chromosome decondensation, which may reflect inherited features from the mother chromosome, in addition to structural impediments to full decondensation.

Labelling single mitotic chromosomes allowed observation of their decondensation. By measuring local fluorescence intensities of a GFP tagged histone H2B in HeLa cells, a previous study measured a decondensation factor of five [17]. For the reverse process, the condensation of chromatin prior to mitosis, an estimated decrease in volume of 2–3 fold was measured, in NRK cells [10]. The same study found an axial shortening after metaphase which peaked 8–12 min after anaphase onset, so the absolute decondensation level may depend upon when the time during mitosis at which the chromosome was labelled and measured. Our technique allows measurement of the decondensation factor for single chromosomes. On average we found decondensation of over 300%, in rough agreement with published data, yet we also detected a high variability in decondensation, ranging from almost zero to over 7 fold. This may reflect the different gene density and transcriptional status of chromosomes [60], [61]. In addition, the obstacles of nuclear compartments, the lamina and other chromosomes have the potential to cause stochastic chromosome-wide effects on measured bulk volume, which is perhaps why we observed sizeable differences in measured volumes between decondensing sister chromatids. This raises the question of whether such a structural impediment could impede gene expression.

Morphological definition of decondensing chromosomes was near complete within the first hour after mitosis (Figure 2b). Beyond this interval, bulk chromosome morphology and volume displayed only incremental changes. This implies the major physical reactions of the nucleus, such as nucleolus formation, space definition of chromosome territories, and establishment of nuclear space with respect to the cytoplasm, have been completed. It would be interesting to observe whether this fixity of position and morphology holds for motile cells, such as neutrophils, which must continuously redefine their cytoskeletons. This may place additional forced dynamicity on interphase chromosome morphology and relative position.

Labelling the chromosomes in mitosis also allows us to compare the daughter chromosomes, conditioned by the same inheritance and cell cycle stages. By eye it was obvious that daughter chromosomes could acquire very different morphologies, and this was supported by volume measurements. Morphology is therefore clearly neither an intrinsic property of chromosomes, nor a completely lineage-dependent one. A simple, perhaps less satisfying hypothesis, is that chromosomes simply fill the space they fall into, which will be defined by interchromosome interactions, chromatin interactions with the lamina and other nuclear compartments, and above all, by the constraints of the cytoplasm on the nucleus. Chromosome architecture will describe the balance between the needs for chromosome decondensation and accessibility with the constraints of the available space.

These balances may also explain the apparent resistance of chromosome architecture to treatments affecting major nuclear functions. Chromosome morphology was not significantly perturbed by blocking transcription, histone deacetylation, ATP synthesis or topisomerase II. These activities have previously been shown to be required for the positioning and dynamics of individual chromosome loci. And we have clearly seen perturbations of nuclear architecture by these treatments in the same cells which fail to lose chromosome structural integrity, in addition to anecdotal effects on morphology of a few chromosomes. These results may explain the ambivalent findings in the literature on the effects of transcription and acetylation on chromatin and chromosome morphology [48], [62], [63], [64], [65], [66]. Physical and functional constraints are likely to be multifactorial, and effects of depleting one or another of these factors will be masked by other stabilising forces. Perhaps more simplistically, physical constraints will mean there are places a single locus can go that a chromosome cannot.

Technical challenges that remain are generic to live cell imaging. How can cells be imaged at high spatial resolution, for long periods of time with minimal bleaching and phototoxicity? Another limitation of our approach, which is generic to many types of imaging, is the method used to define the edge of the chromosome region. Whether by automated script or by eye, there is always a user-defined aspect to thresholding. The existence of “looped-out” chromosome regions implies the threshold applied is generally too severe, with the edge defined to close to the chromosome centroid. How a user is supposed to improve upon current definition, without clear hybridisation or photolabel signal, is at present unclear. It is likely the unknown dispersal beyond the “visible” edge will impact on measurements, in this study and elsewhere.

A further issue with so-called caged fluorescent proteins is their tendency to activate (uncage) under normal illumination. In the present study, we have counteracted these issues by restricting the numbers of 3D stacks we captured after mitosis. This was especially important when carrying out 3 colour imaging, involving detection of fluors with overlapping spectra. Another persistent issue is the use of drugs to stall mitosis, to allow a greater number of chromosomes to be labelled in a given imaging routine. Greater automation of the photoactivation protocol would diminish the need for these treatments.

Live cell technologies provide greater temporal resolution of cellular events than extrapolations from fixed cells. They also provide mechanistic hypotheses not foreseeable from fixed material, or homogenous population extracts. It is easier to understand a process when one can observe the object of ones interest before, during and after a particular process or treatment. The ability to view single chromosome architecture in living cells is imperative to our future understanding of the functional nuclear landscape. This will, in turn, be fuelled by improvements in fluorescent protein photostability and efficiency, detection sensitivity, and automated capture and analysis routines, with the goal of imaging a chromosome and its loci through a whole cell cycle, from decondensation to condensation. This will allow us to begin to address the problem of how a chromosome folds.

## Materials and Methods

### Cell culture and transfection of cells

All cells were grown in DMEM media (Invitrogen) supplemented with 10% fetal bovine serum (Invitrogen). Stable cell lines expressing fluorescent and photoactivateable proteins were established in HT-1080 cells [30], by selection of Lipofectamine 2000-transformed cells. The fusions were expressed from the p3′ss backbone [6] by replacing the GFP-lacI sequence with H2B-mRFP (H2B from [67]) and H3-PA-GFP (HIST1H3A) and YFP-lacI fusions. All fusions were cloned into the XbaI/EcoRV digested backbone. The HT1080 cell with lacO repeats integrated in chromosome region 11q13 was described previously [16]. H2B mRFP and H3 PA-GFP were cotransformed using Attractene (QIAGEN). Cells were selected in 100 µg/ml hygromycin and single colonies were isolated with expression of both proteins. For 11q13 cells, YFP-lacI was introduced in a second transformation round, cotransformed with pcDNA3.1 to utilise its G418 cassette as a selectable marker. A high degree of variegation of YFP-lacI was observed in single clones. To enrich the proportion of YFP-lacI expressing cells, YFP+ve/RFP+ve cells were separated from YFP-ve/RFP+ve by fluorescent activated cell sorting (FACS) using a FACS Vantage cell sorter with DIVA upgrade (Becton Dickinson).

### Live imaging

For live cell imaging cells were grown in the same media but buffered with 25 mM Hepes pH 7 [20]. Cells were grown on glass gridded dishes from IWAKI (#3922-035) or Ibidi (#81166). For treatment with Monastrol, cells were placed 20 h prior to microscopy in imaging dishes. Cells were incubated for 3 h with 100 µM Monastrol [31](Calbiochem), directly before microscopy cells were washed twice with PBS (Invitrogen) and fresh media was added. For synchronizing cells using RO-3306 [37](Calbiochem) the cells were innoculated 36 h before microscopy. Inhibitor was added 16–18 h before at 3.5 µM. After the incubation cells were washed twice in PBS and fresh media was added. After 45 min of recovery, which allowed many cells to enter mitosis, cells were used for photolabelling.

Activation of single chromosomes in mitosis was performed using the LeicaSP2 confocal microscope with a HCX PL APO 63x/1.40–0.60 oil λ_BL_ objective. For activation, 6 scans defined by the Leica ROI tool were carried out at full power with the 405 nm laser line. For evaluating whether only single chromosomes were labelled, a 3D stack (z interval 500 nm, pinhole 1.00AU) of the mitotic cell was captured immediately after activation. The cells were brought back to the incubator after activation and cell division monitored using bright field. Gridded dishes allowed relocation of cells with activated chromosomes. Daughter cells were imaged using an inverted Axiovert 200 microscope (Zeiss) with an imagEM EM-CCD camera (C9100-13, Hamamatsu) and a Zeiss Plan-Apochromat 63x/1.40 oil objective [32], [33], [34]. 3D stacks were imaged with 500 nm z-steps. The system was managed by Volocity Acquisition (Improvision). Images were deconvolved afterwards using Volocity Restoration software. For chromosome 11 labelling, imaging was carried out on the confocal, which allowed proper separation of YFP and GFP. This separation exacerbated bleaching of both fluors, therefore limited 3D stacks were captured after cell division.

Progression through mitosis has been used as a measure of cell health [68]. However, excitation with UV light could lead to DNA damage. To address this issue, we activated nuclei using our labelling protocol then stained samples 2 h after photoactivation with an antibody against phosphorylated H2AX (Calbiochem; DR1017), which marks DNA repair foci. We observed no difference in staining between photolabelled and non-labelled nuclei. A more intensive labelling, using 20 confocal scans rather than 6 also failed to stimulate phospho-H2AX. In contrast, 5Gy of γ irradiation or 50 µM etoposide elicited a strong stimulation of phospho-H2AX.

For drug treatments to inhibit nuclear processes, cells were treated 90 minutes after division for 1 h with 1 µM actD (Sigma Aldrich), 10 nM TrichostatinA (Sigma Aldrich) or 50 µM etoposide (Calbiochem). For ATP depletion daughter cells were incubated for 30 min with 10 mM sodium azide (Sigma Aldrich) and 50 mM 2-deoxyglucose (Sigma Aldrich). Treatment was reversed by washing the cells twice with PBS followed by recovery incubation in standard media for 30 minutes.

### Image Analyses and Visualisation

Image analyses were performed using the Volocity Measurement Software (Improvision). Thresholding and outlining of territories was carried out by the software, with accuracy of chromosome boundaries in each z plane assessed by eye, the most accurate method when dealing with small to medium sized data sets. Volume and surface measurements were calculated directly by Volocity from these data. Volume measurements are voxel counts, surface measurements were derived from the summed area of a skin of triangles fitted to the surface voxels of the data set. Sphericity values were calculated from volume and surface area data. Confocal files were imported in Volocity and analysed the same way. To calculate long axes of chromosomes, images were imported into ImageJ as tiff files and converted to 8 bit. Single chromosomes were defined and thresholded using the plug-in “3D object counter” [69]. Measurements were performed using the “3D manager” function of the “binary morphological filter” plug-in [70]. This plug-in also allowed volumes, surface area and sphericity to be measured. Volume was estimated as the number of pixels in the object multiplied by the volume of one voxel. For surface area was number of border pixels multiplied by surface of one voxel. This alternative analysis revealed comparable results to those estimated by Volocity. Statistical comparisons were carried out using the Wilcoxon signed rank test and Mann-Whitney test for unpaired observations (comparing fixed and live cells).

To assess variability of measurements from the two different microscopes used, 3D images of fixed fluorescent nuclei were captured with both the confocal (mitotic imaging) and widefield (interphase imaging) stations. Volume measured was similar between both microscopes (mean 3% higher by widefield) however surface area was estimated to be 27% higher on the confocal, indicating the apparent mild increase in surface area during decondensation may only be an effect of the imaging protocol.

### Immunofluorescence, Western Blotting and FISH

For measuring the effects of actD on nuclear structure, we stained cells with α-mouse coilin hybridoma supernatant (from Angus Lamond), α-mouse fibrilarrin (Abcam #18380) and α-mouse Ki-67 (BD # 610968). A Cy3-conjugated donkey anti mouse antibody (Jackson #715-165-150) was used as a secondary. For evaluating TSA treatment we probed Western blots of control and TSA-treated extracts of H2B-mRFP/H3-PA-GFP expressing cells with α-mouse anti-GFP (Roche # 11814460001), a α-rabbit H3 acetyl antibody, H3K9Ac and H3K14Ac (all acetylation antibodies from Upstate). Horseradish-coupled anti-rabbit IgG (Sigma Aldrich) or anti-mouse IgG (Biorad) were used as secondary antibodies. Staining protocols were adapted from [71].

For comparing live and fixed cell data FISH was performed using a whole chromosome 11 and a lacO-repeat specific probes [20]. Cells were cultured overnight on SuperFrost slides (VWR) and fixed either as asynchronous populations or after wash-out of RO-3306. Before fixation cells were washed once in PBS, rinsed in CSK buffer (10 mM Pipes pH 6.8, 10 mM NaCl, 300 mM sucrose, 3 mM MgCl and 2 mM EDTA pH 8.0) and were permeablised for 10 min in CSK buffer containing 0.5% Triton X-100. After washing with PBS cells were fixed for 10 min in a 4% buffered paraformadehyde solution (Electron Microscope Sciences # 15713). 3D FISH was then carried out as described [40]. The FISH protocol based upon lysis before fixation was selected as it allows greater probe access with less stringent DNA denaturation.

## Supporting Information

Figure S1 Testing the efficiency of drug treatments A) Reduction of transcription by treatment of HT-1080 cells with 1 µM actinomycinD. HT-1080 cells were cultured overnight on glass coverslips, then treated for one hour with or without 1 µM actinomycinD. Cells were then treated with 2.5 mM 5-fluorouridine (Sigma F5130) for 40 minutes. Cells were then fixed in 4% paraformaldehyde, lysed in 1% triton in PBS, then stained with a mouse anti-BrdU antibody (Sigma B2531) at a dilution of 1 in 500 in PBS with 2% BSA as a block [72]. B) Perturbation of H2B-mRFP staining density in HT-1080 cells treated with 10 mM azide and 50 mM 2-deoxyglucose. C) Induction of a nuclear wide DNA damage response by etoposide treatment. HT-1080 cells were cultured overnight on glass coverslips, then treated for one hour with or without 50 µM etoposide. Cells were then fixed and stained with antisera against phospho-H2AX (see Materials and Methods). Although the untreated controls show some non-specific cytoplasmic staining with the H2AX antibody, uniform nuclear staining is strongly induced by etoposide.
(1.56 MB TIF)
