# Supplementary figures and images for: Stable Morphology, but Dynamic Internal Reorganisation, of Interphase Human Chromosomes in Living Cells
Source: PLoS One. 2010 Jul 13;5(7):e11560. doi: 10.1371/journal.pone.0011560 (PMC2903487; doi:10.1371/journal.pone.0011560)

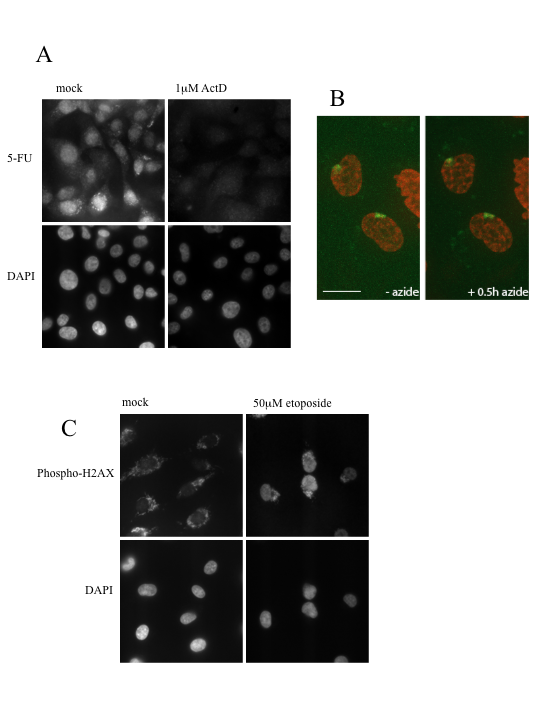

Supplement: Figure S1 — Testing the efficiency of drug treatments A) Reduction of transcription by treatment of HT-1080 cells with 1 µM actinomycinD. HT-1080 cells were cultured overnight on glass coverslips, then treated for one hour with or without 1 µM actinomycinD. Cells were then treated with 2.5 mM 5-fluorouridine (Sigma F5130) for 40 minutes. Cells were then fixed in 4% paraformaldehyde, lysed in 1% triton in PBS, then stained with a mouse anti-BrdU antibody (Sigma B2531) at a dilution of 1 in 500 in PBS with 2% BSA as a block [72]. B) Perturbation of H2B-mRFP staining density in HT-1080 cells treated with 10 mM azide and 50 mM 2-deoxyglucose. C) Induction of a nuclear wide DNA damage response by etoposide treatment. HT-1080 cells were cultured overnight on glass coverslips, then treated for one hour with or without 50 µM etoposide. Cells were then fixed and stained with antisera against phospho-H2AX (see Materials and Methods). Although the untreated controls show some non-specific cytoplasmic staining with the H2AX antibody, uniform nuclear staining is strongly induced by etoposide. (1.56 MB TIF) [file pone.0011560.s001.tif]
